# Influence of maternal age on birth and infant outcomes at 6 months: a cohort study with quantitative bias analysis

**DOI:** 10.1093/ije/dyac236

**Published:** 2023-01-06

**Authors:** Elisabeth Gebreegziabher, Mamadou Bountogo, Ali Sié, Alphonse Zakane, Guillaume Compaoré, Thierry Ouedraogo, Elodie Lebas, Fanice Nyatigo, Maria Glymour, Benjamin F Arnold, Thomas M Lietman, Catherine E Oldenburg

**Affiliations:** Francis I. Proctor Foundation, University of California San Francisco, San Francisco, CA, USA; Department of Epidemiology and Biostatistics, University of California, San Francisco, CA, USA; Centre de Recherche en Santé de Nouna, Nouna, Burkina Faso; Centre de Recherche en Santé de Nouna, Nouna, Burkina Faso; Centre de Recherche en Santé de Nouna, Nouna, Burkina Faso; Centre de Recherche en Santé de Nouna, Nouna, Burkina Faso; Centre de Recherche en Santé de Nouna, Nouna, Burkina Faso; Francis I. Proctor Foundation, University of California San Francisco, San Francisco, CA, USA; Francis I. Proctor Foundation, University of California San Francisco, San Francisco, CA, USA; Department of Epidemiology and Biostatistics, University of California, San Francisco, CA, USA; Francis I. Proctor Foundation, University of California San Francisco, San Francisco, CA, USA; Department of Ophthalmology, University of California, San Francisco, CA, USA; Francis I. Proctor Foundation, University of California San Francisco, San Francisco, CA, USA; Department of Epidemiology and Biostatistics, University of California, San Francisco, CA, USA; Department of Ophthalmology, University of California, San Francisco, CA, USA; Francis I. Proctor Foundation, University of California San Francisco, San Francisco, CA, USA; Department of Epidemiology and Biostatistics, University of California, San Francisco, CA, USA; Department of Ophthalmology, University of California, San Francisco, CA, USA

**Keywords:** Maternal age, adolescent pregnancy, infant outcomes, quantitative bias analysis

## Abstract

**Background:**

Maternal age is increasingly recognized as a predictor of birth outcomes. Given the importance of birth and growth outcomes for children’s development, wellbeing and survival, this study examined the effect of maternal age on infant birth and growth outcomes at 6 months and mortality. Additionally, we conducted quantitative bias analysis (QBA) to estimate the role of selection bias and unmeasured confounding on the effect of maternal age on infant mortality.

**Methods:**

We used data from randomized–controlled trials (RCTs) of 21 555 neonates in Burkina Faso conducted in 2019–2020. Newborns of mothers aged 13–19 years (adolescents) and 20–40 years (adults) were enrolled in the study 8–27 days after birth and followed for 6 months. Measurements of child’s anthropometric measures were collected at baseline and 6 months. We used multivariable linear regression to compare child anthropometric measures at birth and 6 months, and logistic regression models to obtain the odds ratio (OR) of all-cause mortality. Using multidimensional deterministic analysis, we assessed scenarios in which the difference in selection probability of adolescent and adult mothers with infant mortality at 6 months increased from 0% to 5%, 10%, 15% and 20% if babies born to adolescent mothers more often died during the first week or were of lower weight and hence were not eligible to be included in the original RCT. Using probabilistic bias analysis, we assessed the role of unmeasured confounding by socio-economic status (SES).

**Results:**

Babies born to adolescent mothers on average had lower weight at birth, lower anthropometric measures at baseline, similar growth outcomes from enrolment to 6 months and higher odds of all-cause mortality by 6 months (adjusted OR = 2.17, 95% CI 1.35 to 3.47) compared with those born to adult mothers. In QBA, we found that differential selection of adolescent and adult mothers could bias the observed effect (OR = 2.24, 95% CI 1.41 to 3.57) towards the null [bias-corrected OR range: 2.37 (95% CI 1.49 to 3.77) to 2.84 (95% CI 1.79 to 4.52)], whereas unmeasured confounding by SES could bias the observed effect away from the null (bias-corrected OR: 2.06, 95% CI 1.31 to 2.64).

**Conclusions:**

Our findings suggest that delaying the first birth from adolescence to adulthood may improve birth outcomes and reduce mortality of neonates. Babies born to younger mothers, who are smaller at birth, may experience catch-up growth, reducing some of the anthropometric disparities by 6 months of age.

Key Messages

- Babies born to adolescent mothers on average had lower weight at birth and lower weight, shorter length, lower mid-upper arm circumference, lower Length-for-Age Z Score, Weight-for-Age Z Score and Weight-for-Height Z Score at enrolment compared with babies of adult mothers.
- Infants born to adolescent mothers had similar growth outcomes to infants born to adult mothers, suggesting that catch-up growth reduces some of the anthropometric disparities in early childhood.
- Infants born to adolescent mothers had twice the risk of mortality by 6 months of age compared with infants of adult mothers.
- Delaying childbirth from adolescence to adulthood can improve birth outcomes and reduce mortality of neonates.
- Whereas the effect of biases on the point estimate for infant mortality is substantial, under all scenarios correcting for selection bias and unmeasured confounding, the odds of mortality in 6 months was higher for babies born to adolescent mothers compared with adult mothers. Changes in estimates in quantitative bias analysis show the importance of considering the role of biases in examining effects of interest.

## Introduction

Maternal age is increasingly recognized as a predictor of birth outcomes. Studies show that pregnancies in the extremes, at ages <17 and >40 years, are at a higher risk of negative birth outcomes than other age groups.^1^^,^^2^ Although maternal age at childbearing has increased over the last decade,^3^ there is still a substantial proportion of young women who give birth during adolescence, particularly in developing countries. Globally, ∼15% of young girls of age 15–19 years give birth before age 18 years.^4^ Reasons for this include early marriage that subsequently leads to early motherhood. Studies show that the rate of child marriage in low- and middle-income countries increases slowly until age 14 years and accelerates after ages 15, 16 and 17 years.^5^ In addition to planned early child bearing, early child marriage also increases unintended pregnancies and negatively impacts the health of both the mother and child.^6^

Previous studies show that babies born to young mothers have a greater risk of very-pre-term and pre-term delivery, low birthweight, small for gestational age and neonatal mortality.^7^^,^^8^ Low birthweight and poor growth have implications on long-term health.^9^ Being of low birthweight has been linked with subnormal growth, illnesses and neurodevelopmental delays which in some cases can still be apparent during adolescence and later life.^10^^,^^11^ Growth monitoring is also important as the pattern of growth is a marker of a child’s wellbeing.^9^^,^^12^ Children with multiple anthropometric failures such as stunting, underweight and wasting have a disproportionately high risk of having issues with cognitive development, school achievement, economic productivity and mortality.^13^^,^^14^ A study showed that an estimated 42% of child deaths can be attributed to children who are stunted, underweight and wasted or stunted and underweight.^13^ Therefore, a neonate’s birth and growth outcomes are important factors for development, wellbeing and survival.

Although some studies show that young maternal age has negative birth outcomes, there is limited evidence regarding the influence of maternal age on anthropometric measures at birth and within the first few months of life, particularly in developing countries where the rates of adolescent childbearing are high. The effect of maternal age on growth outcomes is also not clear. Whereas some studies show that under-five morbidity may still be higher in children born to younger mothers and that the effect of poor birth outcomes may persist in early childhood,^15^^,^^16^ other studies show that the gap between normal and smaller babies (with low birthweight or low birth length) narrows in the months after birth.^17^ Hence, it is important to examine the short- and long-term effects of maternal age on child birth, growth and mortality outcomes.

Using data from a large randomized–controlled trial of neonates in Burkina Faso,^18^ this study aimed to examine the effect of maternal age on infant birth and growth outcomes at 6 months and mortality. Since this study used data from a trial that enrolled infants who were ≥8 days old and weighed >2.5 kg at enrolment, and did not have a measure of socio-economic status (SES), we conducted deterministic and probabilistic bias analysis for selection bias and unmeasured confounding to assess the effect of these biases on the estimates of the association between maternal age and infant mortality at 6 months.

## Methods

### Study design, setting and population

This analysis used data from a randomized–controlled trial of azithromycin vs placebo conducted to establish the efficacy and safety of administration of a dose of azithromycin during the neonatal period.^18^ The trial recruited 21 832 babies in Burkina Faso in 2019 and 2020. The study took place in several regions of Burkina Faso, including urban, peri-urban and rural areas that are within a 4-h drive of a facility with paediatric surgical capacity because azithromycin may increase the risk of infantile hypertrophic pyloric stenosis, which requires surgical intervention.^18^ Mothers and infants were recruited either during antenatal care visits, via facility births or via a key informant who notified study staff of a birth. Neonates were enrolled between 8 and 27 days of age and had to weigh >2500 g at enrolment. Infants who had low birthweight were included if they weighed >2500 g at enrolment.

### Data collection and measures

At baseline, trained field workers collected information of the infant and mother via a questionnaire. Child information included birthweight, type and timing of breastfeeding initiation and whether the child was born at a health centre. Maternal information collected included age, education level, number of previous pregnancies, number of prenatal care visits, region of residence and pregnancy type (single vs multiple). The vital status of each infant enrolled was assessed by field workers at 28, 90 and 180 days after enrolment. Anthropometric measurements were made at baseline and at 6 months, and included weight in kilograms (kg), length in centimetres (cm) and mid-upper arm circumference (MUAC). Weight was measured using a digital scale that was standardized each morning before measurement. Length was measured using a Shorrboard to the nearest cm. At both visits, three consecutive measurements for length were taken and the median was used for analysis. MUAC was measured using a standard MUAC tape. Birthweight data were extracted from government-issued health cards of babies enrolled in the trial.

For this analysis, maternal age was dichotomized as adolescent (13 to <20 years) and adult (20–40 years). We excluded babies born to mothers for whom maternal age was missing (*n* = 7) and those aged >40 years (*n* = 270). This is because of data-quality issues, as those aged >40 years were in some cases the grandmother of the baby as opposed to the mother and due to the non-linearity in the effect as pregnancies in older age may also result in negative birth outcomes.

Outcomes at enrolment/birth included birthweight (in g), weight (in kg), length (in cm), MUAC (in cm), Length-for-Age Z Score (LAZ), Weight-for-Age Z Score (WAZ) and Weight-for-Length Z Score (WLZ), which are measures of stunting (LAZ < –2 SDs), underweight (WAZ < –2 SDs) and wasting (WLZ < –2 SDs), respectively. All outcomes mentioned above except birthweight were also measured and analysed at 6 months as growth outcomes, i.e. as change from enrolment to 6 months of age. All-cause mortality was determined based on the vital status assessment at 180 days.

### Statistical analysis methods

To assess the effect of maternal age on birth and growth outcomes, we used simple and multivariable linear regression. The changes in outcomes between maternal age groups were expressed as beta coefficients.

Potential confounders of the association between maternal age and birth and growth outcomes and mortality were selected a priori using a directed acyclic graph (DAG)^19^ (Supplementary Figure 1, available as Supplementary data at *IJE* online) and based on previous literature on this topic. Covariates that were adjusted in multivariable models include region (residence in urban vs rural settings) and maternal education. We did not adjust for prenatal care visits as they occur after maternal age and can thus potentially mediate the effect of maternal age on birth and growth outcomes.

**Figure 1:**
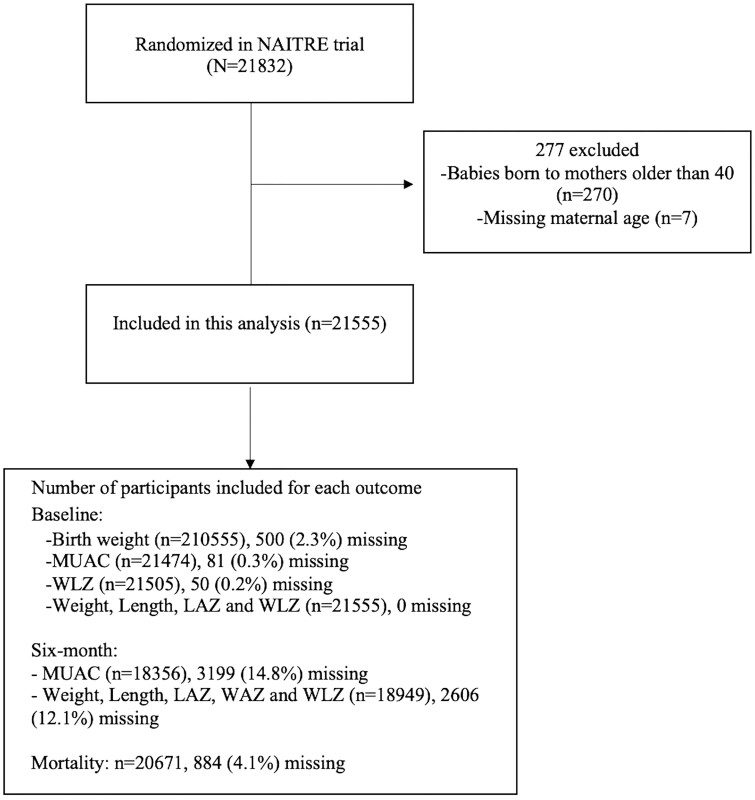
Participant flow diagram. NAITRE, Neonates and Azithromycin, an Innovation in the Treatment of Children in Burkina Faso; MUAC, mid-upper arm circumference; LAZ, Length-for-Age Z Score; WAZ, Weight-for-Age Z Score; WLZ, Weight-for-Length Z Score

To determine the effect of maternal age on mortality, we used logistic regression models with estimates expressed as odds ratios (ORs). Complete case analysis was conducted. For 6-month anthropometry measures, which had some missing outcome values (Figure 1); factors associated with missing values were assessed and sensitivity analyses were conducted using inverse probability censored weighting in which observations were weighted by the inverse of the probability of being observed as determined by the logistic regression model for probability of being observed given covariates and outcome. Covariates included in the logistic regression model were having low birthweight, maternal age, birth order, maternal education, prenatal care visits, residence in urban vs rural settings, child sex and being born at a health centre. As a sensitivity analysis, the association between maternal age and birth and growth outcomes was examined by restricting the data to first-born babies to control for confounding by birth order. The same analyses methods as described above were used for the subgroup analysis. As an additional sensitivity analysis, we examined the effect of maternal age as a continuous variable on childbirth, growth and mortality outcomes. For this analysis, maternal age was rescaled such that each unit increase represented at 5-year increase in maternal age. We conducted another sensitivity analysis in which we adjusted for baseline outcomes to compare estimates that are unadjusted, adjusted only for the known confounders (maternal education and region) and estimates in which confounders as well as potential mediators (baseline measures, birthweight for mortality outcome) are adjusted. This was intended to provide insights into the total effect of maternal age on growth/mortality outcomes as well as the effect it has on the 6-month outcomes beyond or independent of the effect it has on baseline/birth outcomes.

For the quantitative bias analysis (QBA), we used the *episens* command in STATA statistical software (StataCorp, College Station, TX, USA) to conduct multidimensional deterministic and probabilistic analysis to correct for differential selection and unmeasured confounding by SES, respectively.^20^^,^^21^ For the selection bias, through deterministic bias analysis, we assessed scenarios in which there could be differential selection into the study cohort if babies born to adolescent mothers more often died during the first week or were of lower weight and hence did not get selected into the study. This is in line with a previous study that found higher rates of infant mortality among babies born to adolescent mothers.^8^ Therefore, we assumed the probability of selection of adolescent mothers whose child was alive at 6 months (exposed control) and adult mothers whose child was alive at 6 months (unexposed control) to be 1.0, the probability of selection of adult mothers with child mortality at 6 months (unexposed case) of 0.95 and the probability of selection of adolescent mothers with child mortality at 6 months (exposed case) of 0.95–0.75. These corresponded to selection bias factors of 0.95–0.79. For the unmeasured confounding by SES, probabilistic sensitivity analysis through Monte Carlo (random-number-based) simulations with 10 000 replications was used to generate bias-adjusted ORs of maternal age–infant mortality association along with 2.5th and 97.5th simulation limits.^21^ Based on previous studies, we assumed that there could be a higher proportion of adolescent mothers living in poverty compared with adult mothers.^22^ We assumed a prevalence of poverty in adult mothers (unexposed) of 0.2–0.25 and a prevalence of poverty in adolescents (exposed) of 0.2–0.4, both with a uniform distribution. These bias parameters were based on a previous study in which 22% of mothers in the study in Burkina Faso were in Quintile 1 (most poor).^23^ We also assumed a poverty–infant mortality (confounder–outcome) association relative risk (RR) of 2.0–2.7 with a log-normal distribution. This was based on a study that showed proportions of child mortality by socio-economic quantile in six countries in West Africa in which the average of the RRs comparing the proportion of child deaths in the poorest to richest SES quintiles was 2.6.^24^ SAS 9.4 was used for data cleaning and descriptive analyses. STATA version 14.2 was used for the regression models.

## Results

Table 1 shows the characteristics of the study participants. Overall, 21 832 babies were randomized into the NAITRE (Neonates and Azithromycin, an Innovation in the Treatment of Children in Burkina Faso) trial of whom 21 555 were included in this analysis (Figure 1). Among mothers between the ages of 13 and 40 years, 15% were adolescent (13–19 years) and 85% were adults (20–40 years). The average maternal age was 26 years. Among adolescents, 84% had never had a previous pregnancy whereas 37.5% of adults had had three or more previous pregnancies. This was the first pregnancy for 17% of the adults. The majority of the teenagers (59.3%) and adults (60.1%) had had three or four prenatal visits over the course of their pregnancy. Nearly half (45%) of the adolescent mothers and 56% of the adult mothers had no education. Over 99% of the pregnancies were singleton. Overall, 98% of the children were born at a health centre. The majority of the adolescent (73%) and adult mothers (76%) resided in an urban region.

**Table 1:** Characteristics of mothers and infants enrolled in the NAITRE trial in Burkina Faso 2019–2020

Characteristics	Total (*n* = 21 555)	Adolescent (13–19) (*n* = 3202, 14.9%)	Adult (20–40) (*n* = 18 353, 85.1%)
Maternal age in years (mean, SD)	25.9 (5.9)	17.9 (1.1)	27.2 (5.3)
**Baseline measures**			
Birthweight in g (mean, SD)	2997.4 (423.3)	2860.1 (381.8)	3021.4 (425.6)
Weight in kg	3.3 (0.47)	3.2 (0.4)	3.4 (0.5)
Length in cm	50.6 (2.0)	50.1 (1.9)	50.7 (2.0)
MUAC in cm	10.9 (1.1)	10.6 (1.1)	10.9 (1.1)
LAZ	–0.5 (1.2)	–0.8 (1.0)	–0.5 (1.1)
WAZ	–0.6 (0.93)	–0.9 (0.9)	–0.6 (0.9)
WLZ	–0.6 (1.3)	–0.8 (1.3)	–0.6 (1.3)
**Six-month measures**			
Weight in kg (mean, SD)	7.3 (1.0)	7.3 (1.0)	7.3 (1.0)
Length in cm	65.8 (2.6)	65.6 (2.6)	65.8 (2.6)
MUAC	14.1 (1.2)	14.0 (1.1)	14.1 (1.2)
LAZ	–0.5 (1.2)	–0.6 (1.2)	–0.5 (1.2)
WAZ	–0.4 (1.1)	–0.5 (1.1)	–0.4 (1.1)
WLZ	–0.1 (1.3)	–0.1 (1.3)	–0.1 (1.3)
**Mortality**	90 (0.44%)	25 (0.82%)	65 (0.37%)
**Child’s sex at birth**			
Male	10 841 (50.3%)	1590 (49.7%)	9251 (50.4%)
Female	10 714 (49.7%)	1612 (50.3%)	9102 (49.6%)
**Number of previous pregnancies (*n*, %)**			
0	5827 (27.0%)	2686 (83.9%)	3141 (17.1%)
1	4869 (22.6%)	450 (14.1%)	4419 (24.1%)
2	3961 (18.4%)	48 (1.5%)	3913 (21.3%)
3 or more	6895 (32.0%)	16 (0.5%)	6879 (37.5%)
**Number of prenatal visits**			
0	56 (0.3%)	15 (0.47%)	41 (0.20%)
1 or 2	2245 (10.4%)	390 (12.2%)	1855 (10.1%)
3 or 4	12 912 (60.0%)	1895 (59.3%)	11 017 (60.1%)
5 or more	6314 (29.3%)	898 (28.1%)	5416 (29.6%)
**Maternal education level**			
None	11 750 (54.5%)	1446 (45.2%)	10 304 (56.1%)
Primary	3932 (18.3%)	633 (19.8%)	3299 (18.0%)
Secondary	5267 (24.4%)	1114 (34.8%)	4153 (22.6%)
Secondary +	605 (2.8%)	9 (0.3%)	596 (3.3%)
**Pregnancy type**			
Single	21 186 (98.3%)	3184 (99.4%)	18 002 (99.0%)
Multiple	368 (1.7%)	18 (0.6%)	350 (1.9%)
**Child born at health centre**			
Yes	21 073 (97.8%)	3146 (98.3%)	17 927 (97.7%)
**Region**			
Rural	3769 (17.5%)	721(22.5%)	3048 (16.6%)
Urban	16 346 (75.8%)	2338 (73.0%)	14 008 (76.3%)
Peri-urban	1425 (6.6%)	143 (4.5%)	1282 (7.0%)
**Breastfed**			
Yes	21 528 (99.9%)	3196 (99.8%)	18 332 (99.9%)

Excludes missing *n* = 3 for number of previous pregnancies, *n* = 28 for number of prenatal visits and *n* = 15 for region.

NAITRE, Neonates and Azithromycin, an Innovation in the Treatment of Children in Burkina Faso; MUAC, mid-upper arm circumference; LAZ, Length-for-Age Z Score; WAZ: Weight-for-Age Z Score; WLZ, Weight-for-Length Z Score.

At baseline, babies born to adult mothers had a slightly higher birthweight in g, weight in kg, length in cm, MUAC, LAZ, WAZ and WLZ. At 6 months, all these measures were similar among babies born to teenage and adult mothers (Table 1). Among babies born to adolescent mothers, 25 (0.82%) had died by 6 months whereas 65 (0.37%) babies from adult mothers had died within 6 months of life.

In multivariable models, compared with babies born to adult mothers, babies born to adolescent mothers on average weighed 161 g less (95% CI –178.8 to –146.7) at birth and weighed 0.18 kg less (95% CI –0.20 to –0.16), were 0.56 cm shorter (95% CI –0.64 to –0.48) and had 0.29 cm narrower (95% CI –0.33 to –0.25) MUAC at enrolment (Table 2). At baseline, the LAZ, WAZ and WLZ were also 0.30 (95% CI –0.34 to –0.26), 0.38 (95% CI –0.41 to –0.34) and 0.23 (95% CI –0.28 to –0.18) SDs lower for babies born to adolescent mothers, respectively. These results were all similar in bivariate and multivariate models that adjusted for region and maternal education (Table 2).

**Table 2:** Unadjusted and adjusted mean differences and odds ratios of baseline (birth) and 6-month (growth and mortality) outcomes, comparing adult mothers (20–40 years old) with adolescent mothers (13–19 years old)

Adolescent (Adult–ref.)	Unadjusted difference (95% CI)	Adjusted difference (95% CI)
**Baseline outcomes**		
Birthweight in g	–161.3 (–177.2 to –145.4)	–162.8 (–178.8 to –146.7)
Weight in kg	–0.18 (–0.20 to –0.17)	–0.18 (–0.20 to –0.16)
Length in cm	–0.57 (–0.64 to –0.49)	–0.56 (–0.64 to –0.48)
MUAC in cm	–0.30 (–0.34 to –0.26)	–0.29 (–0.33 to –0.25)
LAZ	–0.30 (–0.34 to –0.26)	–0.30 (–0.34 to –0.26)
WAZ	–0.38 (–0.42 to –0.35)	–0.38 (–0.41 to –0.34)
WLZ	–0.24 (–0.29 to –0.19)	–0.23 (–0.28 to –0.18)
**Six-month outcomes**		
Weight in kg	–0.07 (–0.12 to –0.03)	–0.07 (–0.12 to –0.03)
Length in cm	–0.26 (–0.37 to –0.15)	–0.28 (–0.39 to –0.17)
MUAC in cm	–0.08 (–0.13 to –0.03)	–0.07 (–0.12 to –0.03)
LAZ	–0.12 (–0.17 to –0.07)	–0.13 (–0.18 to –0.08)
WAZ	–0.08 (–0.12 to –0.03)	–0.08 (–0.13 to –0.04)
WLZ	–0.01 (–0.06 to 0.04)	–0.002 (–0.06 to 0.05)

Mortality (Adult–ref.)	Unadjusted odds ratio, (95% CI)	Adjusted Odds Ratio, (95% CI)

	*2.24 (1.41 to 3.56)*	*2.17 (1.35 to 3.47)*

Covariates in adjusted models include region and maternal education.

MUAC, mid-upper arm circumference; LAZ, Length-for-Age Z Score; WAZ, Weight-for-Age Z Score; WLZ, Weight-for-Length Z Score.

**Figure 2:**
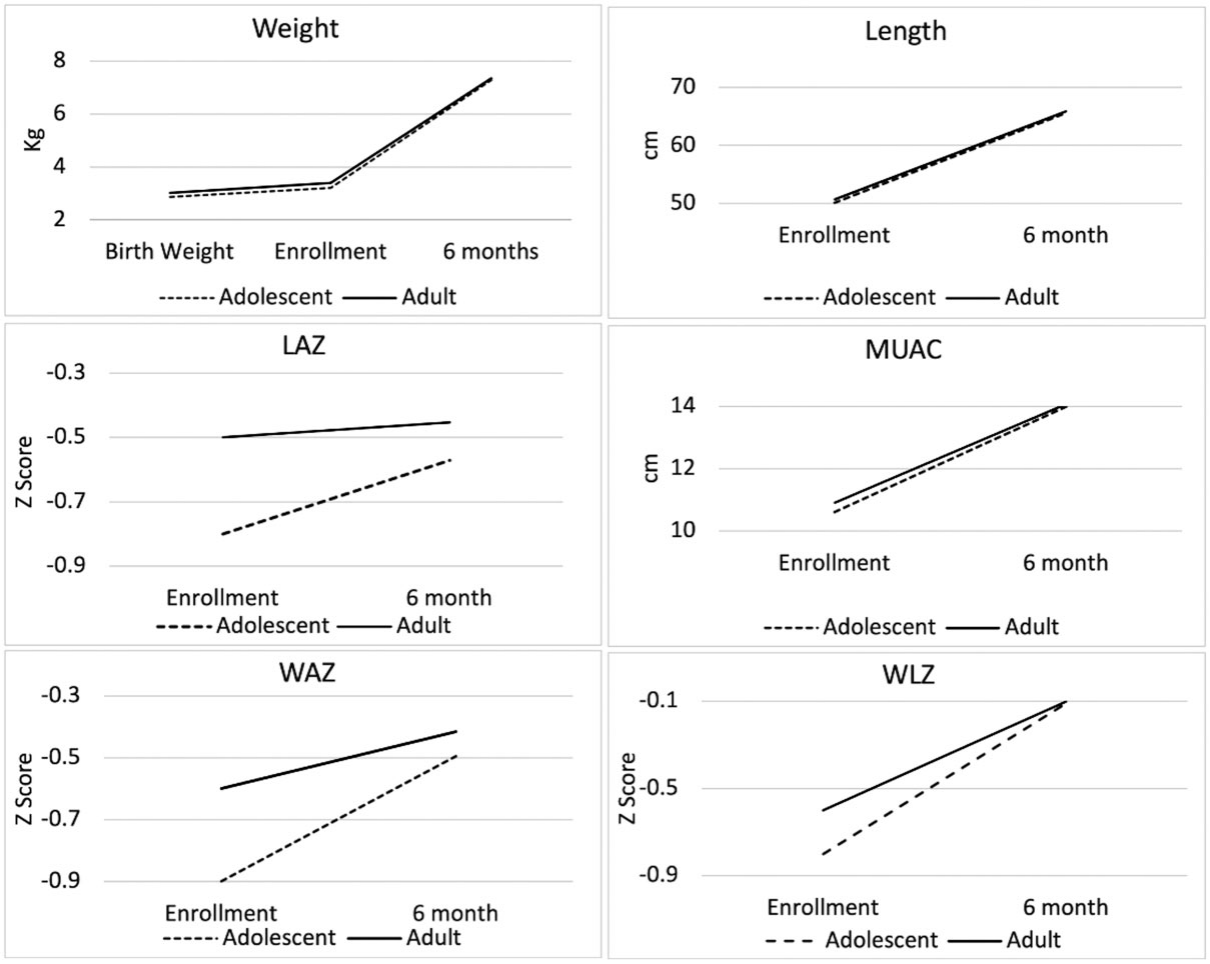
Change in mean anthropometric measures between enrolment and 6 months by maternal age. MUAC, mid-upper arm circumference; LAZ, Length-for-Age Z Score; WAZ: Weight-for-Age Z Score; WLZ, Weight-for-Length Z Score

At 6 months, there were very small decreases in weight, length, MUAC, LAZ and WAZ for babies born to adolescent mothers compared with those born to adult mothers. Overall, at 6 months, there were only narrow and negligible differences in anthropometric measures between babies born to adolescent vs adult mothers (Table 2 and Figure 2). Estimates obtained from inverse probability of censoring weighting for growth outcomes were very similar to complete case analysis from unweighted analyses (Supplementary Table S1, available as Supplementary data at *IJE* online).

In a sensitivity analysis restricting to first-born children (*n* = 5827), the estimates for baseline outcomes were attenuated but similar to the unrestricted analyses except for the difference in length and LAZ, which were no longer substantial. At 6 months, in adjusted models, the mean growth outcomes were consistently lower for babies born to adolescent mothers compared with those born to adult mothers but were not substantial (Supplementary Table S2, available as Supplementary data at *IJE* online). These differences in growth outcomes were negligible.

For the sensitivity analysis using maternal age as a continuous variable, we found that an increase in maternal age was associated with higher/better baseline measures and very small increases in 6-month anthropometric measures (Supplementary Table S3, available as Supplementary data at *IJE* online). For the sensitivity analysis adjusting for baseline measures, we found that including them in the models weakened the association between maternal age and 6-month growth and mortality outcomes, consistently with baseline measures being mediators (Supplementary Table S4, available as Supplementary data at *IJE* online).

For the association between maternal age and infant mortality, in the model adjusted for region and maternal education, babies born to adolescent mothers had 2.17 (95% CI 1.35 to 3.47) times the odds of death by 6 months compared with babies born to adult mothers (Table 2). In sensitivity analysis, restricting to first-born babies did not qualitatively change results (Supplementary Table S2, available as Supplementary data at *IJE* online). In the sensitivity analysis in which maternal age was a continuous variable, each 5-year increase in maternal age was associated with a 21% decrease in the odds of mortality (adjusted OR: 0.79, 95% CI 0.65 to 0.96).

For the QBA, the observed unadjusted OR of the maternal age–infant mortality association was 2.24 (95% CI 1.41 to 3.57). For the selection bias analysis, the bias-corrected (adjusted ORs) for probability of selection of adolescent mothers with child mortality at 6 months (exposed case) of 0.90, 0.85, 0.80 and 0.75 were 2.37 (95% CI 1.49 to 3.77), 2.51 (95% CI 1.58 to 3.99), 2.67 (95% CI 1.67 to 4.24) and 2.84 (95% CI 1.79 to 4.52), respectively (Table 3). The ORs in all scenarios were >1. For the unmeasured confounding bias analysis, the OR corrected for systematic error/confounding was 2.06 (95% CI 1.31 to 2.64), whereas the OR correcting for systematic and random error was 1.98 (95% CI 1.05 to 3.46; Table 4). The bias-corrected OR was closer to the null than the observed OR.

**Table 3:** Multidimensional deterministic sensitivity analysis for selection bias for association between maternal age and child mortality at 6 months

Probability of selection of adolescent mothers with child mortality at 6 months (exposed case)	Probability of selection of adult mothers with child mortality at 6 months (unexposed case)	Selection bias factor	Percent bias	External adjusted odds ratio
1.0	1.0	1.00	0%	2.24 (1.41, 3.57)
0.95	0.95	1.00	0%	2.24 (1.41, 3.57)
0.90	0.95	0.95	–5%	2.37 (1.49, 3.77)
0.85	0.95	0.89	–11%	2.51 (1.58, 3.99)
0.80	0.95	0.84	–16%	2.67 (1.67, 4.24)
0.75	0.95	0.79	–21%	2.84 (1.79, 4.52)

Observed OR = 2.24 (1.41, 3.57).

Assuming 100% probability of selection of adolescent mothers whose child was alive at 6 months (exposed control) and adult mothers whose child was alive at 6 months (unexposed control), 95% probability of selection of adult mothers with child mortality at 6 months (unexposed case) and 95% to 75% probability of selection of adolescent mothers with child mortality at 6 months (exposed case).

Selection bias factor =  *(S_D+E+_/S_D+E–_)/(S_D−E+_/S_D–E–_)*.

**Table 4:** Probabilistic sensitivity analysis for unmeasured confounding by poverty for association between maternal age and infant mortality at 6 months

Analysis	Median	95% Interval (2.5th, 97.5th percentile)
Conventional	2.24	(1.41, 3.57)
Systematic error	2.06	(1.31, 2.64)
Systematic and random error	1.98	(1.05, 3.46)

Assuming prevalence of poverty in adolescents (exposed) of 0.2–0.4, prevalence of poverty in adults (unexposed) of 0.2–0.25 and poverty–infant mortality (confounder–outcome) OR of 2.0–2.7.

## Discussion

In this study, babies born to adolescent mothers had poorer birth and neonatal outcomes as seen in baseline anthropometric measures and a higher risk of all-cause mortality by 6 months but similar growth outcomes at 6 months compared with those born to adult mothers.

Similarly to findings of previous studies, babies born to adolescent mothers had lower weight, height, MUAC, LAZ, WAZ and WLZ at baseline and higher odds of mortality by 6 months.^7^^,^^25–27^ This may partly be due to biological and social mechanisms.^27^^,^^28^ One reason may be physiological/biologic factors related to young motherhood such as short stature, low bodyweight in relation to height, lower maternal body mass index and greater likelihood of inadequate weight gain.^27^ Since younger girls are still growing and physically immature, their nutritional and energy needs may compete with those of the fetus, leading to developmental problems and low-birthweight infants.^29^ Short maternal stature and physical immaturity in adolescence have also been associated with negative birth outcomes such as being small for gestational age, pre-term birth and neonatal mortality.^29^^,^^30^ Additionally, birthweight may be a predictor for wasting and stunting.^31^^,^^32^ Therefore, the low birthweight among infants of young mothers may partially explain the other anthropometric failures. Additionally, behavioural and social factors may also contribute to the poorer outcomes seen among infants of young mothers. These may include lack of maturity, inexperienced childbearing and the higher possibility of pregnancies in adolescence being unplanned and unwanted.^33^^,^^34^ Social factors such as SES may affect access to and adequate utilization of healthcare and medical treatment, information and maternal malnutrition, which may lead to health-related vulnerabilities.^8^ Parental nutritional status and SES have been previously associated with child growth failure.^35^ Older mothers generally have more opportunities and experiences that may allow them to be more educated, financially stable and emotionally mature, which can help in child birth and motherhood.^22^ These factors collectively may contribute to the poorer outcomes seen among infants of adolescent mothers. The reasons noted above may also partly explain the increased risk of mortality among neonates of adolescent mothers. Studies show that adolescence is a risk factor for having a low-birthweight baby and having a low birthweight has been associated with increased risk of mortality not only during infancy, but also in later life.^36^ However, in a separate analysis controlling for birthweight, we still found higher odds of mortality among babies born to adolescent mothers (Supplementary Table S4, available as Supplementary data at *IJE* online). This may again be explained by factors above and beyond birthweight that have previously been associated with risk of infant mortality.^37^

At 6 months, there were very small differences between babies born to adolescent and adult mothers in most anthropometric outcomes in fully adjusted models. This may be due to the smaller babies gaining weight more quickly. Previous studies show that there may be a faster weight-gain ‘catch-up growth’ among infants who are of low birthweight compared with those who are not.^9^^,^^12^ The risk vs benefit of this catch-up growth has been controversial. Accelerated growth in low-birthweight infants can help prevent growth faltering. However, faster weight gain in infancy has been linked to greater risk of obesity and metabolic diseases in later life.^12^^,^^38^ The risk–benefit of post-natal growth may also differ in different populations. In developed countries, promoting catch-up growth particularly through nutritional supplementation may not be advantageous whereas in developing countries, the priority may be supporting growth and preventing malnutrition.^9^^,^^39^ Therefore, whereas the absence of a large difference in growth outcomes at 6 months is a positive finding, the rate of growth should be monitored with regard to the effects it may have in later life. Additionally, a large proportion of the effect of maternal age on growth outcomes may be mediated by birth outcomes. In models in which only maternal education and region of residence are adjusted, babies from adult mothers had slightly greater growth, similar to the trend seen in birth outcomes. However, in the same models, when controlling for baseline measures, the differences became much smaller in magnitude and non-substantial (Supplementary Table S4, available as Supplementary data at *IJE* online). Poor birth outcomes have been previously associated with higher risk of infant mortality and morbidity.^15^^,^^36^ Therefore, differences at 6 months or the lack thereof may be partially explained by the difference in baseline measures.

In sensitivity analysis restricting to first-born children to account for confounding by birth order, our results were mostly similar but attenuated in magnitude. Babies born to adolescent mothers had poorer birth outcomes and higher mortality but similar 6-month growth outcomes. This attenuation of effect may be partly explained by differences related to birth order. Studies show that independently of maternal age, a child’s birthweight increases with birth order.^40^^,^^41^ Since 84% of the newborns among adolescents and only 17% of the infants born to adult mothers were first-born, parity may partially explain some of the differences seen in baseline anthropometric measures of infants born to adolescent vs adult mothers. However, we still found meaningful differences between these two groups in the subgroup analysis of first-born children.

There was potential for selection bias in this study because neonates had to weigh >2500 g (be of sufficient weight) and be ≥8 days old (survive their first week of life) to be enrolled in the trial. If babies born to adolescent mothers more often died during the first week or were of lower weight, this could lead to differential selection into the study cohort. This would bias the results towards the null as a higher proportion of the well (survivors with higher weight) babies from adolescent mothers would be represented in the study. This is similar to our finding from the QBA in which the adjusted OR went further away from the null as the difference in the selection probability of adolescent and adult mothers with child mortality at 6 months increased from 0% to 5%, 10%, 15% and 20%. When considering the causal structure, this is similar to collider stratification in which we restrict the study to babies who survived their first week of life and are of sufficient weight, therefore opening the back-door path from maternal age to infant mortality (Supplementary Figure S2, available as Supplementary data at *IJE* online). This is because being born to an adolescent mother is associated with infant survival in the first week and having a lower weight at birth^7^^,^^8^ and possibly at enrolment, whereas other factors such as SES could also be a cause of low birthweight^42^^,^^43^ and infant mortality.^44^ For the confounding bias analysis, since SES is a predictor of infant mortality and there is likely a higher proportion of adolescent mothers living in poverty compared with adult mothers,^22^ there could be unmeasured confounding. That could mean that part of the maternal age–infant mortality association seen could be due to social and financial disadvantage that is more common among adolescents and leading to the higher infant mortality among this group. This would bias the observed effect away from the null. Our finding also shows that the bias-corrected adjusted OR would be closer to the null compared with the observed OR. Whereas the effect of these biases on the point estimate is substantial, under all scenarios correcting for selection bias and unmeasured confounding, the odds of mortality within 6 months was higher for babies born to adolescent mothers compared with adult mothers.

This study has some limitations. First, there were some missing values for 6-month anthropometric measures/outcomes (12–15%) and babies born to adolescent mothers were more likely to have missing outcomes (15.3% vs 11.5% of those born to adult mothers). However, estimates obtained from inverse probability of censoring weighting for growth outcomes were very similar to complete case analysis from unweighted analyses (Supplementary Table S1, available as Supplementary data at *IJE* online). Second, the generalizability of the findings may be limited to low- to middle-income countries. Third, there were other important factors that were not measured or retrospectively collected such as gestational age at birth, as it required ultrasound, which was not available in the context the study was conducted. Additionally, smoking during pregnancy as well as maternal or paternal height and weight were not measured and may be potential confounders that could contribute to residual confounding.

## Conclusion

Our findings show that delaying the first birth from adolescence to adulthood can improve birth outcomes and reduce mortality of neonates. Delaying pregnancy may allow young girls to mature mentally and physically and improve their social status, autonomy and decision-making, which can lead to better outcomes for their newborns. Additionally, babies born to younger mothers, who are smaller at birth, may experience catch-up growth, reducing some of the anthropometric disparities by 6 months of age. Growth within the first few years of life should be monitored closely to adjust supplements to make up for deficits at birth but not lead to overgrowth, which in turn negatively affects children. Changes in estimates in QBA show the importance of considering the role of biases in examining the effects of interest.

## Ethics approval

The randomized–controlled trial from which the data were obtained was reviewed and approved by the Comité d’Ethique pour la Recherche en Santé (National Research Ethics Committee) in Ouagadougou, Burkina Faso (protocol 2018–10-123) and the University of California, San Francisco Institutional Review Board (protocol 18–25027). Written informed consent was obtained from the caregiver of each enrolled child.

## Supplementary Material

dyac236_Supplementary_Data

## Data Availability

The data for this study are available upon request.
