# Supplementary material for: Influence of maternal age on birth and infant outcomes at 6 months: a cohort study with quantitative bias analysis
Source: Int J Epidemiol. 2023 Jan 6;52(2):414–25. doi: 10.1093/ije/dyac236 (PMC10114123; doi:10.1093/ije/dyac236)
Supplement: dyac236_Supplementary_Data [file dyac236_supplementary_data.docx]

| **Table S1**-Unadjusted and adjusted mean differences of six-month (growth) outcomes with 95% CI using inverse probability of censoring weighting regression analyses | | |
| --- | --- | --- |
| **Adolescent (Adult–ref.)** | **Unadjusted coefficient, (95% CI)** | **Adjusted coefficient, (95% CI)** |
| **Six-month outcomes** |  |  |
| Weight in kg | -0.07 (-0.11 to -0.03) | -0.07 (-0.11 to -0.03) |
| Length in cm | -0.26 (-0.37 to -0.15) | -0.28 (-0.39 to -0.17) |
| MUAC in cm | -0.08 (-0.13 to -0.04) | -0.07 (-0.12 to -0.03) |
| LAZ | -0.12 (-0.16 to -0.07) | -0.13 (-0.18 to -0.08) |
| WAZ | -0.08 (-0.12 to -0.03) | -0.08 (-0.12 to -0.04) |
| WLZ | -0.01 (-0.06 to 0.04) | 0.00 (-0.05 to 0.05) |
| MUAC: mid-upper arm circumference, LAZ: Length-for-Age Z Score, WAZ: Weight-for-Age Z Score, and WLZ: Weight-for-Length Z score.  Covariates in adjusted models include region and maternal education. | | |

| **Table S2-**Sensitivity analyses restricting to first-born infants. Unadjusted and adjusted mean differences and odds ratios of Baseline (birth) & Six-month (growth) outcomes with 95% CI (N=5,827) | | |
| --- | --- | --- |
| **Adolescent (Adult–ref.)** | **Unadjusted coefficient, (95% CI)** | **Adjusted coefficient, (95% CI)** |
| **Baseline outcomes** |  |  |
| Birth weight in grams | -54.8 (-74.9 to -34.7) | -45.2 (-66.2 to -24.2) |
| Weight in kg | -0.06 (-0.08 to -0.03) | -0.04 (-0.07 to -0.02) |
| Length in cm | -0.15 (-0.25 to -0.05) | -0.08 (-0.19 to 0.02) |
| MUAC in cm | -0.20 (-0.26 to -0.14) | -0.17 (-0.23 to -0.11) |
| LAZ | -0.08 (-0.13 to -0.03) | -0.05 (-0.10 to 0.01) |
| WAZ | -0.12 (-0.17 to -0.08) | -0.10 (-0.15 to -0.05) |
| WLZ | -0.09 (-0.16 to -0.02) | -0.10 (-0.17 to -0.03) |
| **Six-month outcomes** |  |  |
| Weight in kg | -0.12 (-0.18 to -0.07) | -0.07 (-0.13 to -0.02) |
| Length in cm | -0.24 (-0.39 to -0.09) | -0.20 (-0.35 to -0.04) |
| MUAC in cm | -0.19 (-0.25 to -0.12) | -0.13 (-0.20 to -0.06) |
| LAZ | -0.10 (-0.17 to -0.03) | -0.09 (-0.16 to -0.02) |
| WAZ | -0.13 (-0.19 to -0.07) | -0.08 (-0.14 to -0.02) |
| WLZ | -0.10 (-0.17 to -0.02) | -0.03 (-0.11 to 0.04) |
| **Mortality (Adult–ref.)** | **Unadjusted Odds Ratio, (95% CI)** | **Adjusted Odds Ratio, (95% CI)** |
|  | *3.41 (1.44 to 8.07)* | *3.36 (1.40 to 8.07)* |
| MUAC: mid-upper arm circumference, LAZ: Length-for-Age Z Score, WAZ: Weight-for-Age Z Score, and WLZ: Weight-for-Length Z score.  Covariates in adjusted models include region and maternal education. | | |

| **Table S3-** Adjusted mean differences and odds ratios of Baseline (birth) & Six-month (growth and mortality) outcomes with each 5 years increase in maternal age | |
| --- | --- |
| **Maternal age (Each unit: 5 years increase)** | **Adjusted difference, (95% CI)** |
| **Baseline outcomes** |  |
| Birth weight in grams | 61.12 (56.26 to 65.98) |
| Weight in kg | 0.05 (0.05 to 0.06) |
| Length in cm | 0.19 (0.17 to 0.21) |
| MUAC in cm | 0.08 (0.06 to 0.09) |
| LAZ | 0.10 (0.09 to 0.11) |
| WAZ | 0.11 (0.10 to 0.12) |
| LAZ | 0.05 (0.03 to 0.06) |
| **Six-month outcomes** |  |
| Weight in kg | 0.01 (0.00 to 0.02) |
| Length in cm | 0.08 (0.05 to 0.11) |
| MUAC in cm | 0.00 (-0.01 to 0.02) |
| LAZ | 0.04 (0.03 to 0.05) |
| WAZ | 0.01 (0.00 to 0.03) |
| WLZ | -0.02 (-0.03 to 0.00) |
| **Mortality (Each unit: 5 years increase)** | **Adjusted Odds Ratio, (95% CI)** |
|  | 0.79 (0.65 to 0.96) |
| MUAC: mid-upper arm circumference, LAZ: Length-for-Age Z Score, WAZ: Weight-for-Age Z Score, and WLZ: Weight-for-Length Z score.  Covariates in adjusted models include region and maternal education. | |

| **Table S4-**Unadjusted and adjusted mean differences and odds ratio of six-month (growth and mortality) outcomes comparing adolescent mothers (13-19y) with adult mothers (20-40y) adjusting for confounders & baseline measures | | | |
| --- | --- | --- | --- |
| **Adolescent (Adult–ref.)** | **Unadjusted difference, (95% CI)** | **^a^ Confounders adjusted Difference, (95% CI)** | **^b^ Confounders & baseline measures adjusted difference, (95% CI)** |
| **Six-month outcomes** |  |  |  |
| Weight in kg | -0.07 (-0.12 to -0.03) | -0.07 (-0.12 to -0.03) | 0.05 (0.01 to 0.09) |
| Length in cm | -0.26 (-0.37 to -0.15) | -0.28 (-0.39 to -0.17) | -0.01 (-0.11 to 0.10) |
| MUAC in cm | -0.08 (-0.13 to -0.03) | -0.07 (-0.12 to -0.03) | 0.04 (0.01 to 0.08) |
| LAZ | -0.12 (-0.17 to -0.07) | -0.13 (-0.18 to -0.08) | -0.01 (-0.05 to 0.04) |
| WAZ | -0.08 (-0.12 to -0.03) | -0.08 (-0.13 to -0.04) | 0.06 (0.02 to 0.11) |
| WLZ | -0.01 (-0.06 to 0.04) | -0.002 (-0.06 to 0.05) | 0.04 (-0.01 to 0.09) |
| **Mortality (Adult–ref.)** | **Unadjusted Odds Ratio, (95% CI)** | **^a^ Confounders adjusted Odds Ratio, (95% CI)** | **^b^ Confounders & birthweight adjusted Odds Ratio, (95% CI)** |
|  | *2.24 (1.41 to 3.56)* | *2.17 (1.35 to 3.47)* | *2.04 (1.26 to 3.30)* |
| MUAC: mid-upper arm circumference, LAZ: Length-for-Age Z Score, WAZ: Weight-for-Age Z Score, and WLZ: Weight-for-Length Z score.  ^a^ Covariates in adjusted models include region and maternal education. | | | |
| ^b^ Covariates for the adjusted model include maternal education, region, and baseline measures (birthweight for mortality outcome). | | | |
|  | | | |

Region

Maternal Education

Birth Order

**Maternal Age**

**Birth outcomes**

**Growth outcomes/Mortality**

Prenatal Care Visits

**Figure S1**- Causal structure for covariate adjustment for assessment of the effect of maternal age on infant six-month growth and mortality outcome

Prenatal care visits and birth outcomes- mediators, maternal education and region-confounders adjusted in models, birth order-confounder adjusted by restriction in sensitivity analyses.

-The same DAG was used for assessment of effect of maternal age on infant birth/baseline outcomes with some modifications in which “birth outcomes” were treated as an outcome (instead of mediator shown above) and growth outcomes were excluded from DAG.

Maternal Age

Socioeconomic Status

Surviving first week of life & >2.5kg at enrollment

Infant Mortality

at 6 months

**Figure S2**- Causal structure for bias analysis in the effect of maternal age on infant mortality
